# Case Report: GABAergic and Serotoninergic Agents for the Treatment and Prevention of Prolonged Dissociative Stupor

**DOI:** 10.3389/fpsyt.2021.641474

**Published:** 2021-05-19

**Authors:** Daisuke Yoshioka, Masaaki Iwata, Koichi Kaneko

**Affiliations:** ^1^Division of Neuropsychiatry, Faculty of Medicine, Tottori University, Yonago, Japan; ^2^Department of Psychiatry, National Hospital Organization Tottori Medical Center, Tottori, Japan

**Keywords:** dissociation, stupor, tonic immobility, freezing, GABA, serotonin, amygdala

## Abstract

Dissociative stupor is a common psychiatric disease lacking an established standard treatment. The lack of therapeutic options may be due to the spontaneous and quick complete remission of most patients. However, since some patients experience multiple relapses and prolonged stupor, investigating potential prevention and treatment options is critical. We reported the case of a 61-year-old Japanese woman who presented with intermittent dissociative stupor for several months. Despite her prolonged symptoms, the administration of lorazepam, escitalopram, and aripiprazole, which selectively enhance GABAergic and serotoninergic activity, improved her stupor and prevented relapse. These findings may help with the treatment of persistent dissociative stupor.

## Introduction

Dissociative stupor is a disease characterized by the reduction or absence of voluntary movements and responsiveness to external stimuli, potentially induced by stress. It is occasionally encountered not only in psychiatry, but also in the emergency department. It is classified as a dissociative disorder according to the International Statistical Classification of Diseases and Related Health Problems (ICD)-10 (F44). Based on prior studies, the prevalence of dissociative stupor and trance is ~85–100 per 1,000; these prior studies remain very scarce. The research Dua conducted has revealed similar results (1). In a study of 55 patients with dissociative disorders, the most common clinical presentation was dissociative stupor (60%) (2). However, Alexander et al. reported that no patient diagnosed with brief dissociative stupor or persistent stupor met the ICD-10 diagnostic criteria for dissociative stupor. Thus, the prevalence of dissociative stupor is quite small (3). According to the ICD-10, the absence of a physical or other psychiatric disorder that could explain the stupor and the presence of clear evidence of recent stressful events are necessary to diagnose the disease. Moreover, genetic, developmental, neurobiological, and psychophysiological studies have supported a model whereby repeated chronic trauma, often in a captivity setting, such as in the case of childhood maltreatment, intimate partner violence (IPV), and/or trafficking experiences, may lead to a preferential freezing/dissociative response to threat (4). Thus, dealing with a patient's stressors is an integral part of the treatment, but it is often difficult to achieve and even sometimes impossible to identify them. Psychological approaches are also used, but patients with dissociative disorders have lower intelligent quotients and socioeconomic statuses (2, 5), thereby decreasing the applicability of these treatments. As such, psychological therapies cannot be applied when a patient has stupor. Furthermore, data on the effectiveness of drugs are limited because the underlying neural mechanisms remain unclear.

Here, we reported a case in which a benzodiazepine alone relieved a patient's nervousness to a certain degree, but the combined administration of escitalopram and aripiprazole was effective in alleviating the prolonged and repeated dissociative stupor and even prevented relapse. A literature review on stupor management was also provided.

## Case Description

A 61-year-old Japanese woman lost consciousness at her office and recovered a few minutes later. Eight days after this episode, she was admitted to a hospital due to the appearance of diverse and fluctuating symptoms, such as sub-stupor, disorganized behavior, and paranoia. She had no relevant medical history. She was the youngest of four siblings. She received average grades in her school days, and she liked sports. She got a job after graduating from high school. Although she got this job, she retired relatively early because of poor social relationships. She got married at the age of 22. She gave birth to and raised three children, to whom she was a good mother. Two years before the current episode, her husband died; consequently, she lived alone. However, her daughter and son lived nearby.

Her physical examination; findings of blood tests, non-contrast-enhanced head computed tomography (CT), and magnetic resonance imaging (MRI) were unremarkable and did not suggest a cause for the symptoms; thus, she was transferred to our hospital for better diagnosis and management. She often laid in her bed with her eyes open and had occasional outbursts of paranoia in which she would ask: “Are you going to kill me?” Because these symptoms were acute at the onset and neither she nor her family had a history of psychiatric illness, we suspected an organic neurological disorder, such as encephalitis. Various tests were conducted to confirm our diagnosis. Indeed, we found intermittent generalized slow-wave activity on electroencephalography (EEG), elevated serum CA19-9 levels (118.3 IU/mL, normal range: < 35 IU/mL), and slightly elevated immunoglobulin G levels (5.8 mg/dL, normal range: < 4.3 mg/dL) in the cerebrospinal fluid. CT and MRI scans were normal.

We administered 400 mg of valproic acid (blood concentration: 65.2 μg/mL) on the 17th day for suspected epilepsy. This was followed by steroid pulse therapy on the 22nd day for suspected autoimmune disease, including paraneoplastic neurological syndrome; however, these treatment options had no effect. No autoantibodies were found, eliminating the possibility of an autoimmune disease. On the 29th day, a carcinoma of the tail of the pancreas was observed on contrast-enhanced CT, and a surgery was scheduled. Although she did not receive treatment that targeted the psychiatric symptoms directly, they improved spontaneously and gradually. A few days before the surgery, the psychiatric symptoms were almost fully recovered (around the 60th day). The pancreatic tumor resection was performed on the 67th day, and her postoperative stay was uneventful. The psychiatric symptoms disappeared, and she was discharged on the 92nd day under a continuous treatment with 400 mg of valproic acid. The cause of the psychiatric symptoms could not be identified.

Following discharge, she led an active life at her daughter's house without any issues or new psychiatric events. On the 110th day, the gastroenterologist explained to her that she had a life expectancy of <2 years based on the intraoperative pathological findings. The next day, she presented with stupor again—in the form of a similar episode as that during the first admission—and she was readmitted to our hospital.

**Figure 1:**
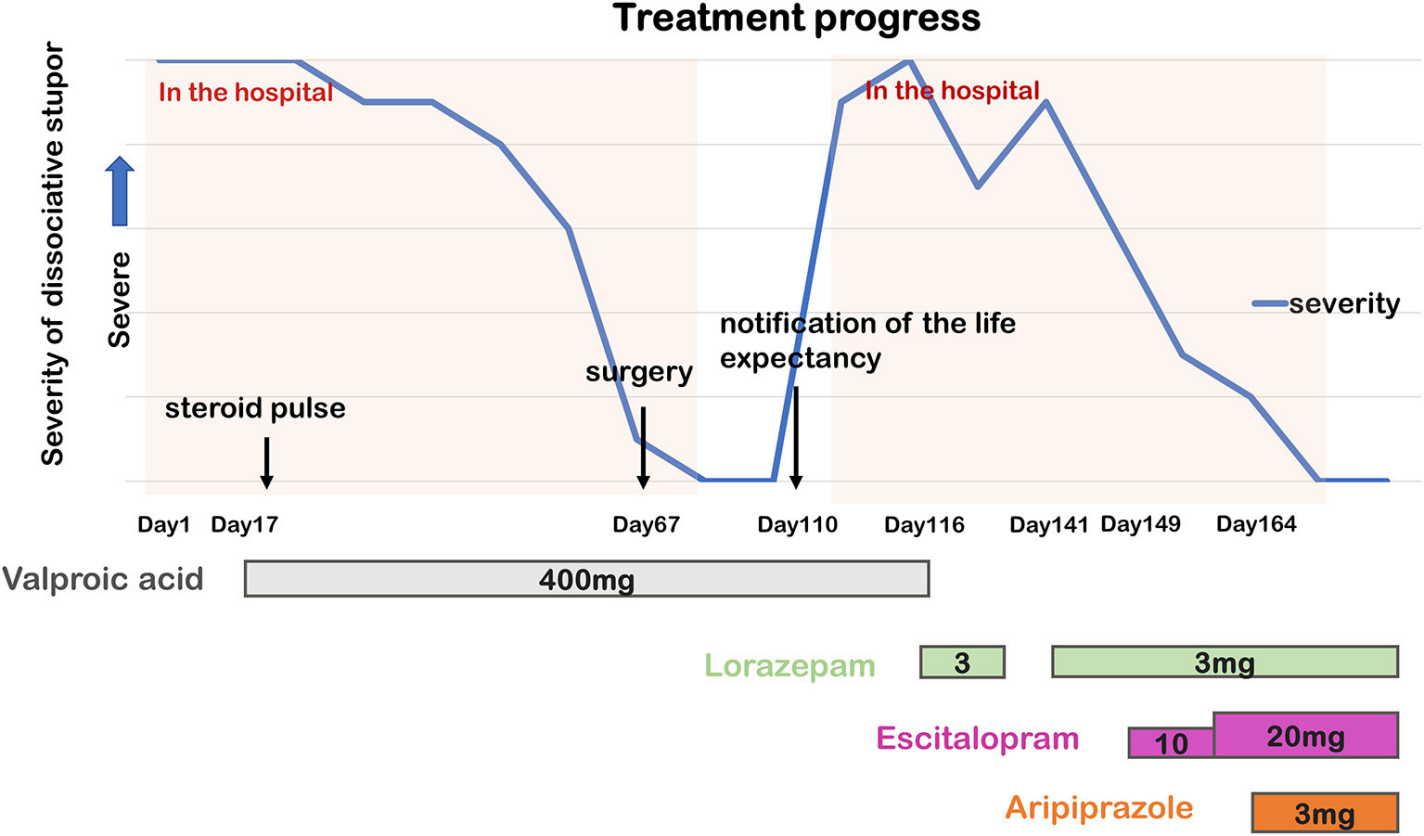
Timeline of the course of dissociative stupor and psychopharmacological treatment. The higher the point on the graph, the greater the stupor severity.

On second admission, head CT and single-photon emission computed tomography (SPECT) scans were performed, but no abnormalities were found. Based on the previous clinical presentation and this one, together with the clear psychological stress, normal physical examination, and absence of abnormal imaging (head CT and MRI, SPECT, and EEG) and laboratory workup findings, a diagnosis of dissociative stupor was made. Furthermore, a relative mentioned that she had stupor for several weeks at the age of 32 when her husband turned down her religious invitation, which improved spontaneously without any medical management. We found out later that her religious beliefs meant that she was against medical treatment. Her hospitalization led to thorough medical help, which may have contributed to the relapse and prolongation of the stupor. To relieve her anxiety, we remained considerate of her feelings; however, the stupor persisted, which made the psychological management challenging. Since valproic acid was ineffective in preventing stupor, we stopped valproic acid administration and initiated treatment with 3 mg of lorazepam to relieve her nervousness on the 116th day. Her symptoms partially improved following this treatment, and we observed her playing table tennis. However, soon afterward, she fell into a stupor again. Eventually, she could not eat at all, and oral treatment was interrupted. Since her condition could not be stabilized and lorazepam alone was insufficient to relieve her anxiety and nervousness, we administered 3 mg of lorazepam again via a nasogastric tube. On the 149th day, we initiated 10 mg of escitalopram to reduce her anxiety. After a week, we increased the dose of escitalopram to 20 mg. At this point, her symptoms partially improved, and she talked more frequently, but she still fell repeatedly and suddenly into a stupor. Since treatment with escitalopram partially improved her stupor, we speculated that medication acting on the serotonergic system would be a more effective treatment. The psychiatric symptoms disappeared a few days after adding 3 mg of aripiprazole to her treatment. She was released from the hospital seven months after the initial hospitalization. We may have refrained from pursuing the inducement in detail, considering her short life expectancy. However, even after the psychiatric symptoms disappeared completely, she had no memory of the situation just before and in the middle of the stupor. The presence of amnesia also supported the diagnosis of dissociative stupor. Despite the antitumoral treatment and palliative care for cancer recurrence, her psychiatric state remained stable until her death from pancreatic cancer a year and 3 months after discharge (Figure 1).

## Discussion

Stupor has several causes, such as organic disorders, including symptomatic mental disorders, schizophrenia, mood disorders, and dissociation disorders. Although these causes cannot be accurately differentiated, some features of dissociative stupor have been reported (Box 1). We first suspected an organic psychological disorder, including paraneoplastic neurological syndrome, as the cause of stupor in this patient. This diagnosis was supported by the acute onset of symptoms, absence of any relevant medical history, or an abnormal physical examination. However, stupor recurred after she was informed of her poor prognosis by the surgeon. The second stupor episode had a clear trigger, and she presented with symptoms similar to those of the first episode. Based on these, dissociative stupor was diagnosed. Furthermore, the information provided by her relative further confirmed our diagnosis. However, we were unable to identify any previous chronic trauma, which, as reported by Loewenstein et al. is a risk for dissociative disorders. Depression was suspected, but it was unlikely due to the sudden onset and her intermittent joyful behavior, which was seen when she played table tennis in between the stupor episodes. Regarding slow-wave electroencephalogram findings in this patient, Kozlowska et al. reported a variety of EEG abnormalities during stupor (9), suggesting that the diagnosis of dissociative stupor cannot be ruled out by the presence of a slow-wave electroencephalogram. However, we must admit a certain discrepancy in this respect, since there were no EEG abnormalities during the second stupor episode.

Box 1 **Features of dissociative stupor**

- It usually occurs in a situation of stress, and superficial motives may be discerned (6).
- The onset is sudden, and the clinical picture changes abruptly with periods of normalcy in between (7).
- Compared to other stupors, dissociative stupor is more likely to wax and wane. There may be a marked emotional reaction when sensitive subjects are discussed (6).
- The patient may show signs of irritability and annoyance when moved against his/her wishes (6).
- There are no hallucinations or delusions at any stage, and there is no evidence of syndromal mood disorder (7).
- No other psychotic syndrome could be clearly identified after the resolution of catatonia (8).

As described in the introduction, no established treatment has been proposed. However, in general, the anxiety at the source of the stupor is sought to be eradicated. We also tried to alleviate her anxiety by interviewing her frequently and asking her family to visit her a lot. However, since she spent most of her time in silence, it was difficult for us to implement psychotherapy. In addition, the fact that she had to undergo surgery despite her devotion to a religion which opposes medical treatment, and that her life expectancy was limited, even though she had been informed of it, must have placed a heavy psychological burden on her. She gradually grew unable to eat, which meant that she needed an urgent medical intervention.

Few studies have focused on dissociative stupor, and its underlying neural mechanism remains unclear. Existing evidence supports conceptualizing dissociation as the human equivalent of the animal “freeze” or “feigning death” protective response in the face of life-threatening danger, where the fight-or-flight response has failed or would be more dangerous (10). Kozlowska et al. classified the defense cascades to fear into four groups (arousal, fight-or-flight, freezing, and tonic immobility [TI]). They stated that freezing is a fight-or-flight response put on hold, and TI or collapsed immobility are last resort responses to an inescapable threat when the active defense responses have failed (9). Further, each of these defense reactions has a distinctive neural pattern mediated by a common neural pathway: the activation and inhibition of functional components in the amygdala, hypothalamus, periaqueductal gray (PAG), and sympathetic and vagal nuclei (9). Referring to the TI characteristics, as summarized by Kozlowska et al., some aspects may apply to this case, including the slow-wave electroencephalogram recordings. Thus, dissociative stupor and TI may be similar to other pathological conditions.

**Figure 2:**
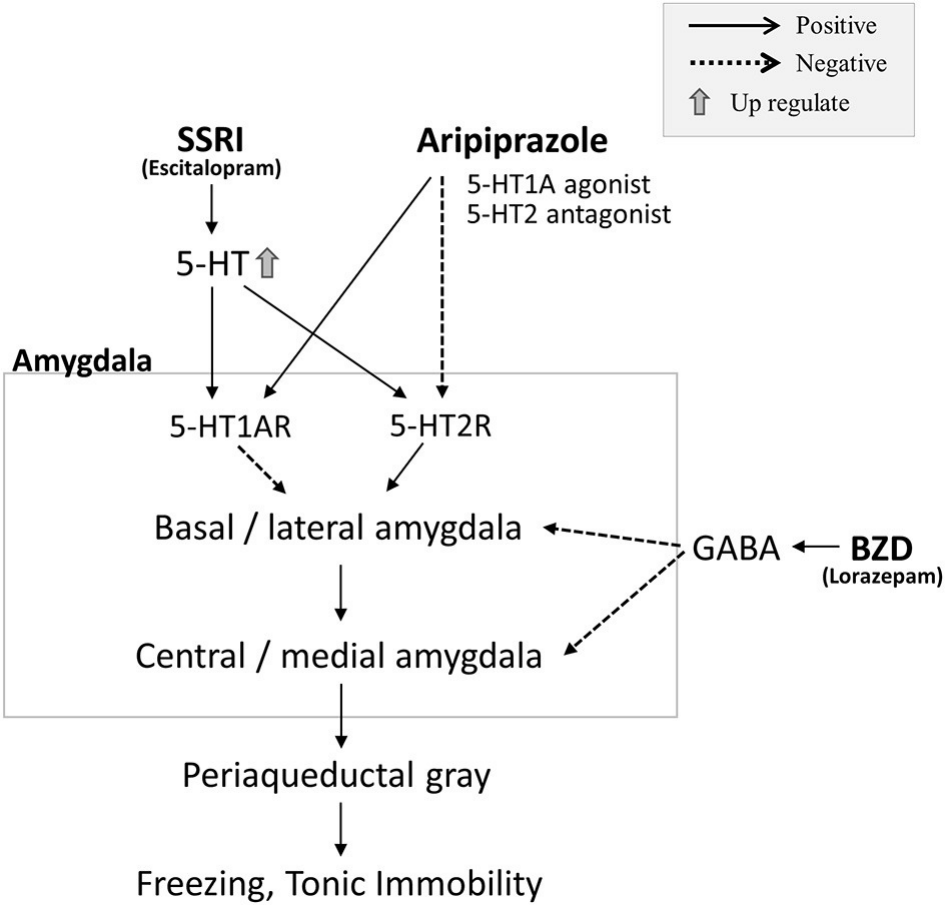
In preclinical studies, stimulation of 5-hydroxytryptamine (5-HT)_1A_ receptors and inhibition of 5-HT_2_ receptors in the amygdala suppressed tonic immobility (TI). Conversely, inhibition of 5-HT1_A_ receptors and stimulation of 5-HT_2_ receptors exacerbated TI. In our case, we hypothesized that the benzodiazepine, lorazepam, would have an inhibitory effect on the amygdala through activation of GABA. A partial effect on stupor was observed with lorazepam; however, the stupor was not completely suppressed. The selective serotonin reuptake inhibitor (SSRI), escitalopram, stimulates both 5-HT_1A_ and 5-HT_2_ receptors, increasing 5-HT concentration; however, this treatment was also insufficient for treating the stupor. When aripiprazole (5-HT_1A_ agonist and 5-HT_2_ antagonist) was administered, the stupor improved, and no relapse of stupor was observed, despite the subsequent stress state. Thus, combined 5-HT_1A_ receptor activation and 5-HT_2_ receptor inhibition through aripiprazole administration may have been effective in treating stupor in this patient.

To the best of our knowledge, no experiments on dissociative stupor have been performed in humans, but many animal experiments on TI have been reported (9). In laboratory settings, we can reproduce TI under simultaneous restraint and fear conditions—for example, turning the animal upside down and restraining it until it stops struggling (9). In experiments replicating TI using similar methods, injections of a GABA_A_ agonist, muscimol, and GABA_B_ agonist, baclofen, into the medial nucleus of the amygdala (MEA) reduced the TI response, while intra-MEA injections of the GABA_A_ antagonist, bicuculine, and GABA_B_ antagonist, baclofen, increased it (11). To date, no effective treatment for stupor has been established, but treatment with benzodiazepines can lead to improvements, as shown in several case reports (12); lorazepam had a partial effect in our patient. In our patient, treatment with escitalopram and aripiprazole had positive effects. Selective serotonin reuptake inhibitors (SSRIs) affect the serotonin system, but aripiprazole, for instance, also acts as a 5-hydroxytryptamine (5-HT)_1A_ receptor agonist and 5-HT_2A_, _2B_, and _7_ receptor antagonist (13). Several reports show that the serotonin system is involved in TI. For instance, activation of 5-HT_1A_ receptors or blockade of 5-HT_2_ receptors in the MEA decreased TI duration in guinea pigs. In contrast, blocking 5HT_1A_ receptors or activating 5-HT_2_ receptors in the amygdala increased the duration of TI (14) (Figure 2). It must be noted that two of the three confirmed stupors resolved spontaneously; moreover, this is a single case report, thus remaining rather speculative. However, in our patient, we can assume that in addition to the inhibitory effects of benzodiazepines in the amygdala, the dissociative stupor might be suppressed by the 5HT_1A_ agonist and 5-HT_2A_ antagonist. Escitalopram stimulates the 5-HT_1A_ and 5-HT_2_ receptors; however, the inhibition of 5-HT_2_ receptors by aripiprazole may have contributed to the extra improvement in the disease state (Figure 2).

Freezing, a defense cascade, can be produced by stimulating the dorsal PAG. Several studies have documented that the administration of benzodiazepines and SSRIs attenuated the fear-like response against the stimulation of dorsal PAG (15, 16). Although freezing and TI are different, they share some similarities; in addition, the elevated freezing threshold mechanism might be involved in preventing stupor reoccurrence (Figure 2). We focused on the GABAergic and serotoninergic systems in this case, but there are reports on the involvement of the dopaminergic system in freezing (17).

This case report remains limited in that aripiprazole may have also influenced the dopaminergic system, and the role of the dopamine system in freezing and TI remains unclear. In addition, the amygdala may be largely involved in the pathological mechanism underlying TI, and the administration of lorazepam, escitalopram, and aripiprazole may have acted on brain regions other than the amygdala. The biological mechanism of dissociative stupor cannot be explained by this case alone and further research is needed. However, since there are very few reports documenting pharmacotherapy for dissociative stupor, this case may shed light on its pathogenesis.

In summary, we suggest that a disturbance in the balance of GABAergic and serotoninergic systems might represent one of the pathophysiologic mechanisms of dissociative stupor. However, since a case report cannot mention the pathophysiology and treatment of dissociative stupor, more cases must be documented.

## Data Availability Statement

The original contributions presented in the study are included in the article/supplementary material, further inquiries can be directed to the corresponding author/s.

## Ethics Statement

Ethical review and approval was not required for the study on human participants in accordance with the local legislation and institutional requirements. The patients/participants provided their written informed consent to participate in this study. Written informed consent was obtained from the individual(s) for the publication of any potentially identifiable images or data included in this article.

## Author Contributions

DY managed the patients, designed the case report, and wrote the manuscript. MI contributed to the writing of the manuscript. KK reviewed the manuscript. All authors contributed to the article and approved the submitted version.

## Conflict of Interest

The authors declare that the research was conducted in the absence of any commercial or financial relationships that could be construed as a potential conflict of interest.
